# The use of dose-escalated radiation for locally advanced non-small cell lung cancer in the U.S., 2004–2013

**DOI:** 10.1186/s13014-016-0755-y

**Published:** 2017-01-17

**Authors:** John P. Christodouleas, Matthew D. Hall, Marjorie A. van der Pas, Wensheng Guo, Timothy E. Schultheiss, Peter Gabriel

**Affiliations:** 1 Department of Radiation Oncology, Hospital of the University of Pennsylvania, 3400, Civic Center Blvd, Philadelphia, 19104 PA USA; 2 Elekta Inc., Atlanta, GA USA; 3 Department of Radiation Oncology, City of Hope National Medical Center, Duarte, CA USA; 4 Department of Biostatistics and Epidemiology, University of Pennsylvania, Philadelphia, PA 19104 USA

## Abstract

**Purpose/Objectives:**

The clinical effects of radiation dose-intensification in locally advanced non-small cell lung (NSCLCa) and other cancers are challenging to predict and are ideally studied in randomized trials. The purpose of this study was to assess the use of dose-escalated radiation for locally advanced NSCLCa in the U.S., 2004–2013, a period in which there were no published level 1 studies on dose-escalation.

**Materials/Methods:**

We performed analyses on two cancer registry databases with complementary strengths and weaknesses: the National Oncology Data Alliance (NODA) 2004–2013 and the National Cancer Database (NCDB) 2004–2012. We classified locally advanced patients according to the use of dose-escalation (>70 Gy). We used adjusted logistic regression to assess the association of year of treatment with dose-escalated radiation use in two periods representing time before and after the closure of a cooperative group trial (RTOG 0617) on dose-escalation: 2004–2010 and 2010–2013. To determine the year in which a significant change in dose could have been detected had dose been prospectively monitored within the NODA network, we compared the average annual radiation dose per year with the forecasted dose (average of the prior 3 years) adjusted for patient age and comorbidities.

**Results:**

Within both the NODA and NCDB, use of dose-escalation increased from 2004 to 2010 (*p* < 0.0001) and decreased from 2010 to 2013 (*p* = 0.0018), even after controlling for potential confounders. Had the NODA network been monitoring radiation dose in this cohort, significant changes in average annual dose would have been detected at the end of 2008 and 2012.

**Conclusions:**

Patterns of radiation dosing in locally advanced NSCLCa changed in the U.S. in the absence of level 1 evidence. Monitoring radiation dose is feasible using an existing national cancer registry data collection infrastructure.

## Introduction

Incremental technical advances in linear accelerators have steadily improved the delivery of radiotherapy to a patient’s tumor while sparing the adjacent normal tissue. Leveraging advances in technology to dose-intensify is compelling particularly in diseases for which local control outcomes are poor, such as inoperable locally advanced non-small cell lung cancer (NSCLCa) which has an estimated 2-year local failure rate of 30% when treated with concurrent chemotherapy and standard dose radiation [1]. However, the clinical effects of dose-intensification in NSCLCa and other cancers have been challenging to predict and several clinical studies have failed to validate presumed benefits [1–3]. A recent example is RTOG 0617 which randomized inoperable locally advanced NSCLCa patients to 60 Gy (standard arm) or 74 Gy (dose-escalation arm) with concurrent chemotherapy [4]. Unexpectedly, patients treated on the dose-escalation arm had substantially worse median overall survival (20.3 months versus 28.7 months), despite having acceptable radiation treatment plans based on established normal tissue constraints.

We hypothesized that because the theoretical rationale for dose-intensified treatments are compelling, they are slowly adopted even in the absence of high level clinical evidence and that this “radiation dose creep” may unexpectedly be causing harm. To assess the first of these hypotheses, that radiation dose creep occurs, we evaluated the patterns of radiation dosing in locally advanced NSCLCa between 2004 and 2013, a period in which there were no published level 1 studies on dose-escalation. We hypothesized that there would be an increasing trend in the use of dose-escalated radiation in the years preceding the closure of RTOG 0617 and that there would be a decreasing trend beginning immediately preceding the study’s closure to the end of the study period.

Finally, we sought to determine the year in which a significant change in radiation dosing practice patterns could have been detected in this study period had dose been prospectively monitored using commonly available cancer registry data.

## Methods

### Data sources

The primary analyses of this study were performed on data extracted from two cancer registry databases with complementary strengths and weaknesses: the National Oncology Data Alliance® (NODA) (Elekta Inc., Sunnyvale, CA), years 2004–2013, and the National Cancer Database (NCDB) (American College of Surgeons, Chicago, Il), years 2004–2012. The NODA captures newly diagnosed cancer cases at more than 150 hospitals in the U.S. and includes all of the data fields sent to state and federal cancer registries. The strength of the NODA is that it includes radiation dose fields that are assessed for internal validity by reviewers with specialized radiation oncology training. The NODA, however, may not be broadly representative of U.S. practice. To assess the generalizability of our observations, we performed the same analysis using data from the NCDB, a more representative database that captures information from approximately 70% of all newly diagnosed cancers in the U.S. [5]. A weakness of the NCDB is that radiation dose is not secondarily validated. Both the NODA and the NCDB require a minimum set of database fields to be completed by registrars to meet submission requirements, so every case of locally advanced NSCLCa treated at participating institutions is not necessarily captured within these databases.

### Cohort identification

To minimize confounding, we sought to restrict our patterns of care analysis to patients likely meeting eligibility criteria for RTOG 0617 since this represents a cohort deemed potentially suitable for dose-escalated radiation by subject matter experts in the era of interest. Using manual chart review as the gold standard, we iteratively developed a cancer registry-based algorithm that classifies patients based on RTOG 0617 eligibility and whether they were treated with definitive-intent concurrent chemotherapy and radiation. To minimize misclassification errors, the algorithm was more restrictive than RTOG 0617 eligibility criteria with respect to tumor staging and prior allowable malignancies. The study cohorts included only patients with AJCC versions 6 and 7 clinical T3, N1 or T0-3, N2, M0, but excluded patients with derived AJCC v6 stage IIB, which included clinical T2, N1, and M0. The algorithm excluded patients treated to total doses of <59 Gy (to omit patients who received pre-operative radiation or palliative radiation or who discontinued radiation early) and patients with total doses of >80 Gy (to omit outliers likely related to reporting errors). We assessed the algorithm’s ability to predict RTOG 0617 eligibility (positive predictive value) in validation cohorts at cancer registry programs in two regionally distinct hospitals.

### Internal and external validity

To assess internal validity (e.g., confounding and bias) within the NODA cohort, we used chi-square tests to compare the distribution of covariates between patients treated in the years before (2004–2010) and after (2011–2013) the early closure of RTOG 0617 (on June 17, 2011). We evaluated the external validity (e.g., generalizability) of our NODA findings in two ways. First, we used chi-square tests to compare the distribution of patient covariates in the NODA cohort (excluding 2013 cases) and the NCDB 2004–2012 cohort. In order to maximize the representativeness of the NCDB, we did not filter the NCDB cohort on radiation dose (unfiltered NCDB 2004–2012) for this first generalizability analysis. Second, after our *a priori* hypotheses were confirmed in the NODA cohort, we determined whether these patterns were also present in the NCDB, 2004–2012, which was similarly filtered on radiation dose (filtered NCDB 2004–2012).

### Primary outcome and control variables

The primary outcome was the use of dose-escalated radiation over two *a priori*-defined periods. Period 1 was defined from 1/1/2004 to 12/31/2010, the period before the early closure of the high dose arms of RTOG 0617 was announced to participating institutions. Period 2 was defined from 1/1/2010 to 12/31/2013, which is the period immediately before RTOG 0617 closure through the data collection period. We defined dose-escalated radiation as a total dose of >70 Gy, consistent with guidelines and on-going national trials [6, 7]. Both NODA and NCDB capture delivered dose, not prescribed dose. We *a priori* selected patient and disease control variables that might affect a physician’s perception of the tolerability and effectiveness of dose-escalated radiation and characteristics of the diagnosing hospital that might affect patterns of care (Table 1). Characteristics of the diagnosing hospital were defined as previously described [8, 9]. We calculated confidence intervals around the estimated annual percentage of patients treated with dose-escalated radiation using the Clopper-Pearson method. We used logistic regression to assess the covariate-adjusted association of the year of treatment (continuous variable) with dose-escalated radiation use (categorical variable) and in each period of interest. In a *post hoc* analysis, we characterized the use of total doses in the ‘low-standard dose’ range (≥59 Gy and <64 Gy), a dose most consistent with the superior arm of RTOG 0617.

**Table 1:** Patient and hospital characteristics before and after 1/1/2011. The closure of the high dose arms of RTOG 0617 was announced on June 17, 2011

Study sample characteristics	NODA 2004–2010 (*N* = 1290)	NODA 2011–2013 (*N* = 443)	*P* value*
	No. (%)	No. (%)	
Age			
≤ 67 yo	663 (51.4)	230 (51.9)	0.85
> 67 yo	627 (48.6)	213 (48.1)	
Sex			
Male	750 (58.1)	269 (60.7)	0.91
Female	540 (41.9)	174 (39.3)	
Race			
White	1127 (87.4)	375 (84.6)	0.12
Non-white	152 (11.8)	68 (14.7)	
Missing	11 (0.8)	3 (0.7)	
Histology			
Squamous	519 (40.2)	226 (51.0)	<0.0001
Non-Squamous	771 (59.8)	217 (49.0)	
Derived AJCC v6 T-stage^a^			
T0-2	862 (66.8)	292 (65.9)	0.42
T3	392 (30.4)	146 (33.0)	
Missing	36 (2.8)	5 (1.1)	
Charlson/Deyo Score			
0	863 (66.9)	195 (60.7)	0.04
1	319 (24.7)	96 (28.0)	
2+	108 (8.4)	34 (11.3)	
Hospital type			
Community	1047 (81.2)	374 (84.4)	0.12
Academic	243 (18.8)	69 (15.6)	
Hospital setting			
Metro	1065 (82.6)	378 (85.3)	0.17
Non-metro	225 (17.4)	65 (14.7)	

*Chi-square tests; missing data was excluded from comparisons. ^a^T–stage comparison was done only for patients staged according to AJCC version 6. Hospital characteristics are those of the diagnosing hospital, not treating institution

We used linear regression to determine the year in which a significant change in radiation dosing could have been detected had total radiation dose been prospectively monitored within the NODA network. We compared the actual average radiation dose (continuous variable) used for a given year across the network with a forecasted average dose based on the average of the prior three years, with adjustments for age and comorbidities. For 2005 and 2006, where three years of prior data were not available, we forecasted based on the prior year and two years, respectively.

Statistical significance was set *a priori* at 0.01 because multiple hypotheses were being tested. Missing values were uncommon so were excluded from statistical analyses.

## Results

The positive predictive values for the RTOG 0617 eligibility algorithm were 90.2% (37/41 patients) and 91.2% (31/34) based on manual chart review at the first and second cancer registry programs, respectively. The most common reason for false positives within both validation sets was failure to meet RTOG 0617 performance status and pulmonary function test criteria. The positive predictive values of the algorithm with respect to total radiation dose were 97.5% (40/41) and 97.1% (33/34) at the first and second cancer registry programs, respectively.

**Fig. 1:**
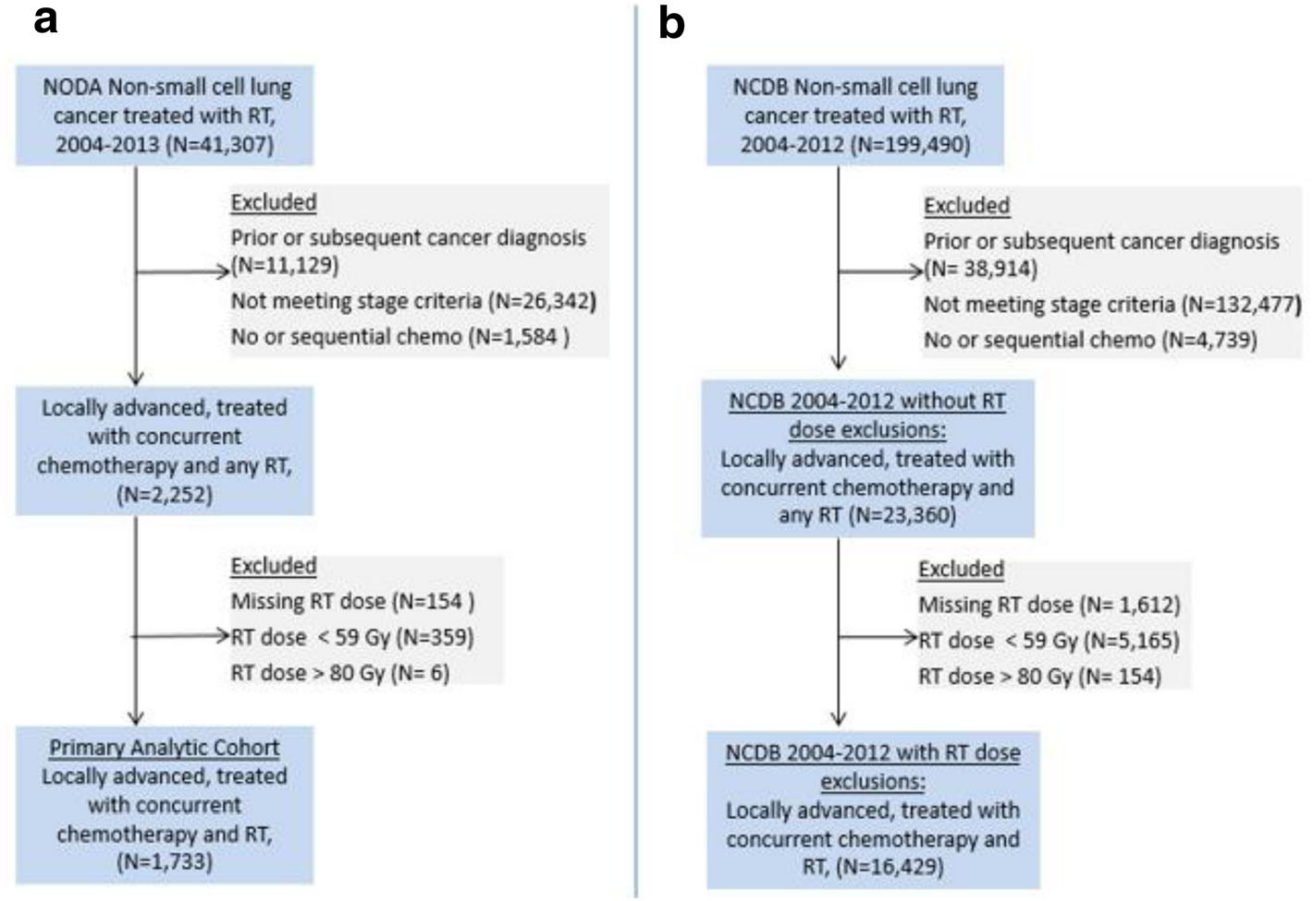
The NODA (**a**) and the NCDB (**b**) cohorts and reasons for exclusions. *Abbreviations: chemo, chemotherapy; RT, radiation; Gy, Gray*

When applied to the NODA dataset, the algorithm identified 1733 patients treated between 2004 and 2013, of whom 499 (29%) patients were treated with dose-escalated radiation (Fig. 1). Table 1 shows the distribution of patient, disease and hospital factors in the period from 2004 to 2010 versus 2011–2013. The patients treated in 2004–2010 and 2011–2013 were similar with respect to age, sex, race, T-stage and diagnosing hospital academic status and metropolitan status. Table 2 shows a comparison of the distribution of patient, disease and contextual variables of the NODA primary analytic cohort (excluding 2013 data) and the NCDB 2004–2012 NSCLCa cohort without radiation dose exclusions (Fig. 1). The NODA cohort had a greater proportion of metropolitan and non-academic hospitals and borderline differences with respect to age, race, T- stage and comorbidity scores.

**Table 2:** Patient and hospital characteristics for the NODA primary analytic cohort (excluding 2013 data) and the NCDB cohort without RT dose exclusions

Study sample characteristics	NODA 2004–2012^a^	NCDB 2004–2012 without RT dose exclusions^a^	*P* value*
	No. (%)	No. (%)	
Age			
≤ 67 yo	829 (51.5)	12,619 (54.0)	0.05
> 67 yo	781 (48.5)	10,741 (46.0)	
Sex			
Male	943 (58.6)	13,422 (57.5)	0.38
Female	667 (41.4)	9938 (42.5)	
Race			
White	1395 (86.6)	19,841 (84.9)	0.03
Non-white	202 (12.5)	3377 (14.5)	
Missing	13 (0.8)	142 (0.6)	
Histology			
Squamous	681 (42.3)	9680 (41.4)	0.50
Non-Squamous	929 (57.7)	13,680 (58.6)	
T-stage^b^			
T0-2	1074 (66.7)	15,418 (66.0)	0.06
T3	499 (31.0)	7942 (34.0)	
Missing	37 (2.3)	0 (0%)	
Charlson/Deyo Score			
0	1063 (66.0)	14,695 (62.9)	0.03
1	400 (24.9)	6209 (26.6)	
2+	147 (9.1)	2456 (10.5)	
Hospital type^c^			
Community	1316 (81.7)	17,207 (73.7)	<0.0001
Academic	294 (18.3)	6153 (26.3)	
Hospital setting^c^			
Metro	1338 (83.1)	17,204 (73.6)	<0.0001
Non-metro	272 (16.9)	5210 (22.3)	
Missing	0 (0)	946 (4.0)	

^a^The NODA cohort was defined by year of treatment start and the NCDB cohort was defined by year of diagnosis. The NODA cohort was filtered using RT dose criteria but the NCDB cohort was not in order to preserve its representativeness of the national lung cancer population. *Chi-square test. Missing data was excluded from Chi-Square comparison. ^b^T –stage comparison was done only for patients staged according to AJCC version 6 and missing data was excluded from Chi-Square comparison. ^c^Hospital characteristics are those of the diagnosing hospital, not treating institution

**Fig. 2:**
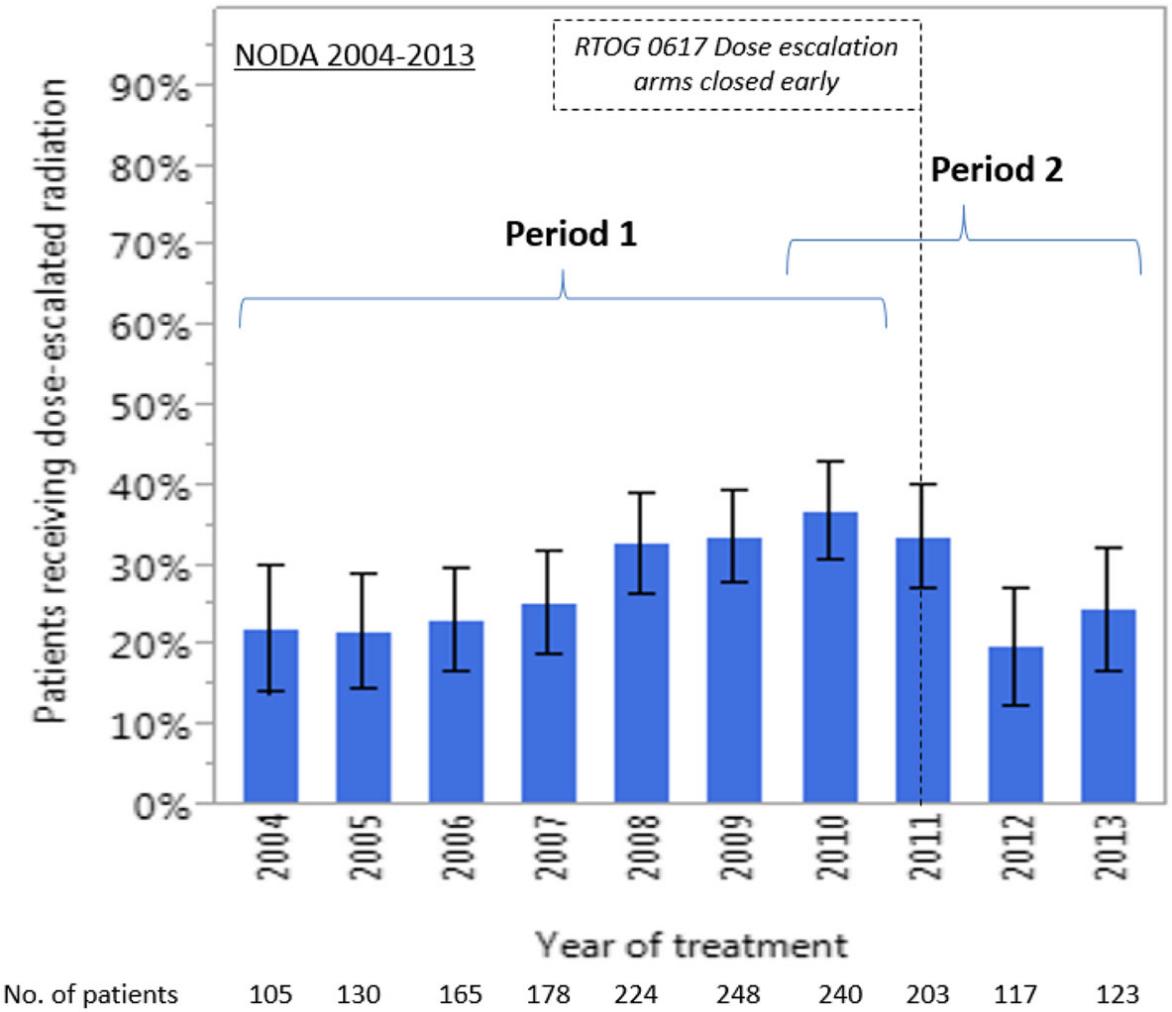
Percentage of locally advanced non-small cell lung cancer patients receiving dose-escalated RT (>70 Gy) over time from 2004 to 2013 in the NODA. Period 1: 1/1/2004 to 12/31/2010. Period 2: 1/1/2010 to 12/31/2013. Bars represent 95% confidence interval

From 2004 to 2010 (Period 1), the use of dose-escalated radiation increased (*p*< 0.0001), even after adjusting for potential confounders (Table 3). From 2010 to 2013 (Period 2), the use of dose-escalated radiation decreased (*p* = 0.0018) (Table 4), even after adjusting for potential confounders. Specifically, the percentage of patients treated with dose-escalated radiation in the NODA increased from 22% (95% CI: 15-31%) in 2004 to 37% in 2010 (95% CI: 31-43%) and then declined to 20% (95% CI: 13–28%) in 2012, its lowest level in the decade (Fig. 2). Because RTOG 0617 was opened by 484 institutions and accrued 544 patients, we sought to determine whether accrual on to the trial itself might explain the apparent change in patterns of care in the NODA dataset. Of the 499 patients treated with dose-escalated radiation in the NODA dataset, only 20 (4%) patients were treated to 74 Gy during the period in which the high dose arms of RTOG 0617 were accruing.

**Table 3:** Logistic regression of dose escalation status and treatment year adjusting for potential confounders in NODA during Period 1 (2004–2010)

	NODA 2004–2010 (*N* = 1408)			
Study sample characteristics	Escalated dose	Standard dose	Escalated vs standard dose	
	No. (%)	No. (%)	Adjusted OR (95% CI)	P*
Treatment year			1.16 (1.09–1.25)	<0.0001
Age				
≤ 67 yo	197 (52)	446 (49)	ref	
> 67 yo	181 (48)	466 (51)	0.92 (0.72–1.18)	0.52
Sex				
Male	230 (61)	520 (57)	ref	
Female	148 (39)	392 (43)	0.82 (0.64–1.06)	0.14
Race				
White	339 (90)	788 (86)	ref	
Non-white	37 (10)	115 (13)	0.61 (0.40–0.90)	0.01
Missing	2 (<1)	9 (1)	Not included	
Histology				
Squamous	168 (44)	351 (38)	ref	
Non-Squamous	210 (56)	561 (62)	0.85 (0.66–1.10)	0.20
Derived AJCC v6 T-stage				
T0-2	263 (70)	599 (66)	ref	
T3	110 (29)	282 (31)	0.87 (0.66–1.14)	0.30
Missing	5 (1)	31 (3)	Not included	
Charlson/Deyo Score				
0	237 (63)	662 (68)	ref	
1	109 (29)	210 (24)	1.45 (1.09–1.93)	0.01
2+	32 (8)	76 (8)	1.10 (0.69–1.73)	0.46
Hospital type^a^				
Community	278 (74)	769 (84)	ref	
Academic	100 (26)	143 (16)	2.33 (1.70–3.20)	<.0001
Hospital setting^a^				
Metro	302 (80)	763 (84)		
Non-metro	76 (20)	149 (16)	1.62 (1.16–2.24)	0.0046

*Chi-square test. Missing data was excluded from Chi-Square comparison. ^a^Hospital characteristics are those of the diagnosing hospital, not treating institution

**Table 4:** Logistic regression of dose escalation status and treatment year adjusting for potential confounders in NODA during Period 2 (2010–2013)

	NODA 2010–2013 (*N* = 567)			
Study sample characteristics	Escalated dose	Standard dose	Escalated vs standard dose	
	No. (%)	No. (%)	Adjusted OR (95% CI)	P*
Treatment year			0.78 (0.66–0.91)	0.0018
Age				
≤ 67 yo	112 (54)	239 (50)	ref	
> 67 yo	97 (46)	235 (50)	0.86 (0.61–1.21)	0.39
Sex				
Male	129 (62)	274 (58)	ref	
Female	80 (38)	200 (42)	0.81 (0.57–1.14)	0.23
Race				
White	181 (86)	394 (82)	ref	
Non-white	28 (13)	80 (17)	0.70 (0.42–1.12)	0.14
Missing	2 (1)	3 (1)	Not included	
Histology				
Squamous	111 (53)	228 (48)	ref	
Non-Squamous	98 (47)	246 (52)	0.79 (0.56–1.12)	0.18
Derived AJCC v6 T-stage				
T0-2	144 (69)	313 (66)	ref	
T3	65 (31)	154 (33)	0.88 (0.61–1.26)	0.49
Missing	0 (0)	7 (1)	Not included	
Charlson/Deyo Score				
0	132 (63)	294 (62)	ref	
1	56 (27)	127 (27)	0.99 (0.67–1.46)	0.98
2+	21 (10)	53 (11)	0.93 (0.52–1.61)	0.80
Hospital type^a^				
Community	160 (77)	405 (85)	ref	
Academic	49 (23)	69 (15)	1.96 (1.27–3.49)	0.0047
Hospital setting^a^				
Metro	174 (80)	412 (87)	ref	
Non-metro	35 (20)	62 (13)	2.07 (1.20–3.01)	0.0024

*Chi-square test. Missing data was excluded from Chi-Square comparison. ^a^Hospital characteristics are those of the diagnosing hospital, not treating institution

**Fig. 3:**
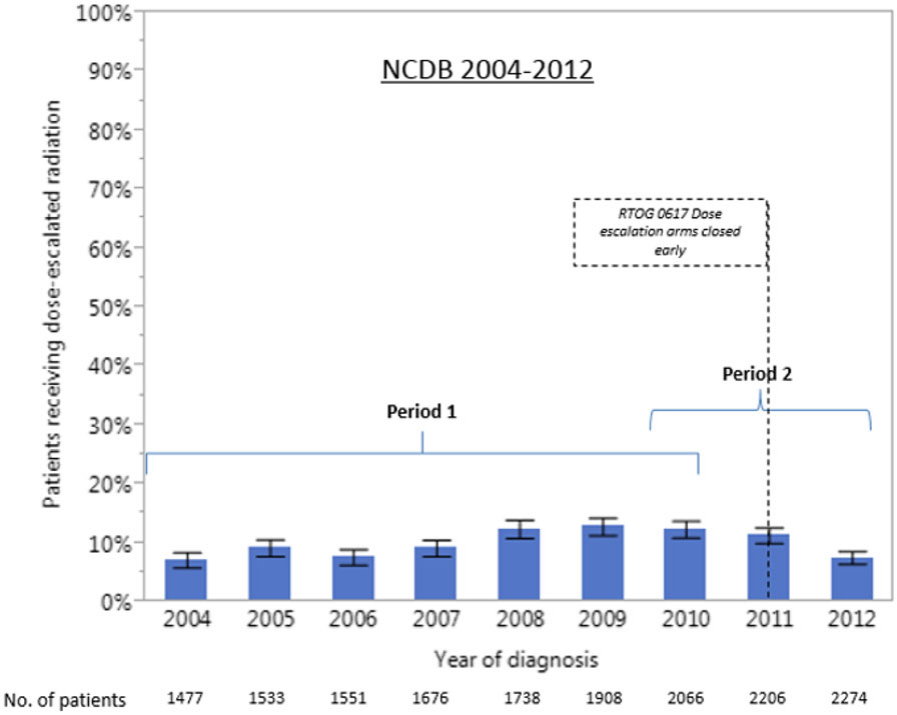
Percentage of locally advanced non-small cell lung cancer patients receiving dose-escalated RT (>70 Gy) over time from 2004 to 2012 in the NCDB. Period 1: 1/1/2004 to 12/31/2010. Period 2: 1/1/2010 to 12/31/2013. Bars represent 95% confidence interval

**Fig. 4:**
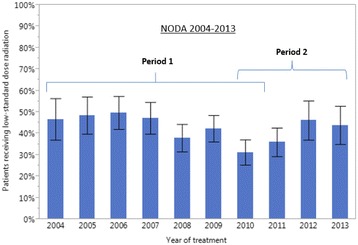
Percentage of locally advanced non-small cell lung cancer patients receiving low-standard dose radiation (<64 Gy) over time from 2004 to 2013 in the NODA. Period 1: 1/1/2004 to 12/31/2010. Period 2: 1/1/2010 to 12/31/2013. Bars represent 95% confidence interval

**Fig. 5:**
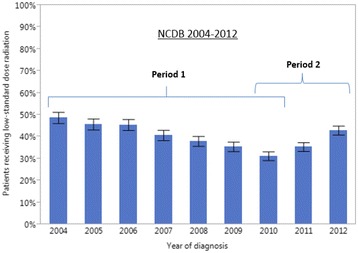
Percentage of locally advanced non-small cell lung cancer patients receiving low-standard dose radiation (<64 Gy) over time from 2004 to 2012 in the NCDB. Period 1: 1/1/2004 to 12/31/2010. Period 2: 1/1/2010 to 12/31/2013. Bars represent 95% confidence interval

An increasing utilization of dose-escalated radiation in Period 1 and a decreasing utilization in Period 2 was also observed in the NCDB 2004–2012 (Fig. 3, Tables 5 and 6). While the pattern of utilization was similar between the two datasets, the absolute estimates of utilization were lower in the NCDB. In the NCDB dataset, the percentage of patients treated with dose-escalated radiation increased from 7% (95% CI: 6–8%) in 2004 to 12% (95% CI: 11-14%) in 2010 then decreased to 7% (95% CI: 6–9%) in 2012. The pattern of utilization of low-standard dose radiation (≥59 and <64Gy) over time was similar in the NODA and NCDB (Figs. 4 and 5, respectively). In the NODA, the percentage of patients treated using low-standard dose RT decreased from 47% (95% CI: 37-56%) in 2004 to its lowest levels of 31% (95% CI: 25–37%) in 2010 and then increased again to 44% (95%CI: 35-53%) in 2013. In the NCDB, the percentage of patients treated using low-standard dose radiation decreased from 49% (95% CI: 46–52%) in 2004 to its lowest level of 31% (95% CI: 29-33%) in 2010 and then increased again to 43% (95% CI: 41–45%) in 2012.

**Table 5:** Logistic regression of dose escalation status and treatment year adjusting for potential confounders of the NCDB cohort with RT dose exclusions during Period 1 (2004–2010)

	NCDB 2004–2010 (*N* = 11,949)			
Study sample characteristics	Escalated dose	Standard dose	Escalated vs standard dose	
	No. (%)	No. (%)	Adjusted OR (95% CI)	P*
Treatment year			1.11 (1.08–1.25)	<0.0001
Age				
≤ 67 yo	708 (57)	5856 (55)	ref	
> 67 yo	524 (43)	4861 (45)	0.89 (0.79–1.01)	0.07
Sex				
Male	689 (56)	6223 (58)	ref	
Female	543 (44)	4494 (42)	1.10 (0.97–1.25)	0.12
Race				
White	1062 (86)	9143 (85)	ref	
Non-white	162 (13)	1497 (14)	0.91 (0.76–1.09)	0.31
Missing	8 (1)	77 (1)	Not included	
Histology				
Squamous	498 (40)	4333 (40)	ref	
Non-Squamous	734 (60)	6384 (60)	1.04 (0.89–1.15)	0.82
Derived AJCC v6 T-stage				
T0-2	826 (67)	7286 (68)	ref	
T3	406 (33)	3431 (32)	1.03 (0.91–1.18)	0.60
Charlson/Deyo Score				
0	786 (64)	6916 (64)	ref	
1	320 (26)	2778 (64)	0.98 (0.85–1.13)	0.81
2+	126 (10)	1023 (10)	1.05 (0.85–1.29)	0.64
Hospital type^a^				
Community	901 (73)	7991 (75)	ref	
Academic	331 (27)	2726 (25)	1.09 (0.95–1.25)	0.23
Hospital setting^a^				
Metro	904 (73.4)	7777 (73)	ref	
Non-metro	274 (22.2)	2476 (23)	0.96 (0.83–1.11)	0.59
Missing	54 (4.4)	464 (4)	Not included	

*Chi-square test. Missing data was excluded from Chi-Square comparison. ^a^Hospital characteristics are those of the diagnosing hospital, not treating institution

**Table 6:** Logistic regression of dose escalation status and treatment year adjusting for potential confounders of the NCDB cohort with RT dose exclusions during Period 2 (2010–2012)

	NCDB 2010–2012 (*N* = 6546)			
Study sample characteristics	Escalated dose	Standard dose	Escalated vs standard dose	
	No. (%)	No. (%)	Adjusted OR (95% CI)	P*
Treatment year			0.77 (0.69–0.85)	<0.0001
Age				
≤ 67 yo	367 (54)	3124 (53)	ref	
> 67 yo	308 (46)	2747 (47)	0.93 (0.79–1.09)	0.38
Sex				
Male	393 (58)	3308 (56)	ref	
Female	282 (42)	2563 (44)	0.94 (0.79–1.11)	0.45
Race				
White	594 (88)	4937 (84)	ref	
Non-white	78 (12)	895 (15)	0.76 (0.59–0.98)	0.04
Missing	3 (<1)	39 (1)	Not included	
Histology				
Squamous	315 (47)	2658 (45)	ref	
Non-Squamous	360 (53)	3213 (55)	0.97 (0.82–1.14)	0.72
Derived AJCC v6 T-stage				
T0-2	425 (63)	3693 (63)	ref	
T3	250 (37)	2178 (37)	1.01 (0.85–1.19)	0.95
Charlson/Deyo Score				
0	406 (60)	3510 (60)	ref	0.80
1	196 (29)	1684 (29)	1.00 (0.83–1.20)	0.83
2+	73 (11)	677 (11)	0.96 (0.73–1.24)	
Hospital type^a^				
Community	520 (77)	4220 (72)	ref	
Academic	155 (23)	1651 (28)	0.80 (0.66–0.97)	0.02
Hospital setting^a^				
Metro	495 (73)	4421 (75)	ref	
Non-metro	163 (24)	1285 (22)	1.07 (0.88–1.29)	0.51
Missing	17 (3)	165 (3)	Not included	

*Chi-square test. Missing data was excluded from Chi-Square comparison. ^a^Hospital characteristics are those of the diagnosing hospital, not treating institution

**Fig. 6:**
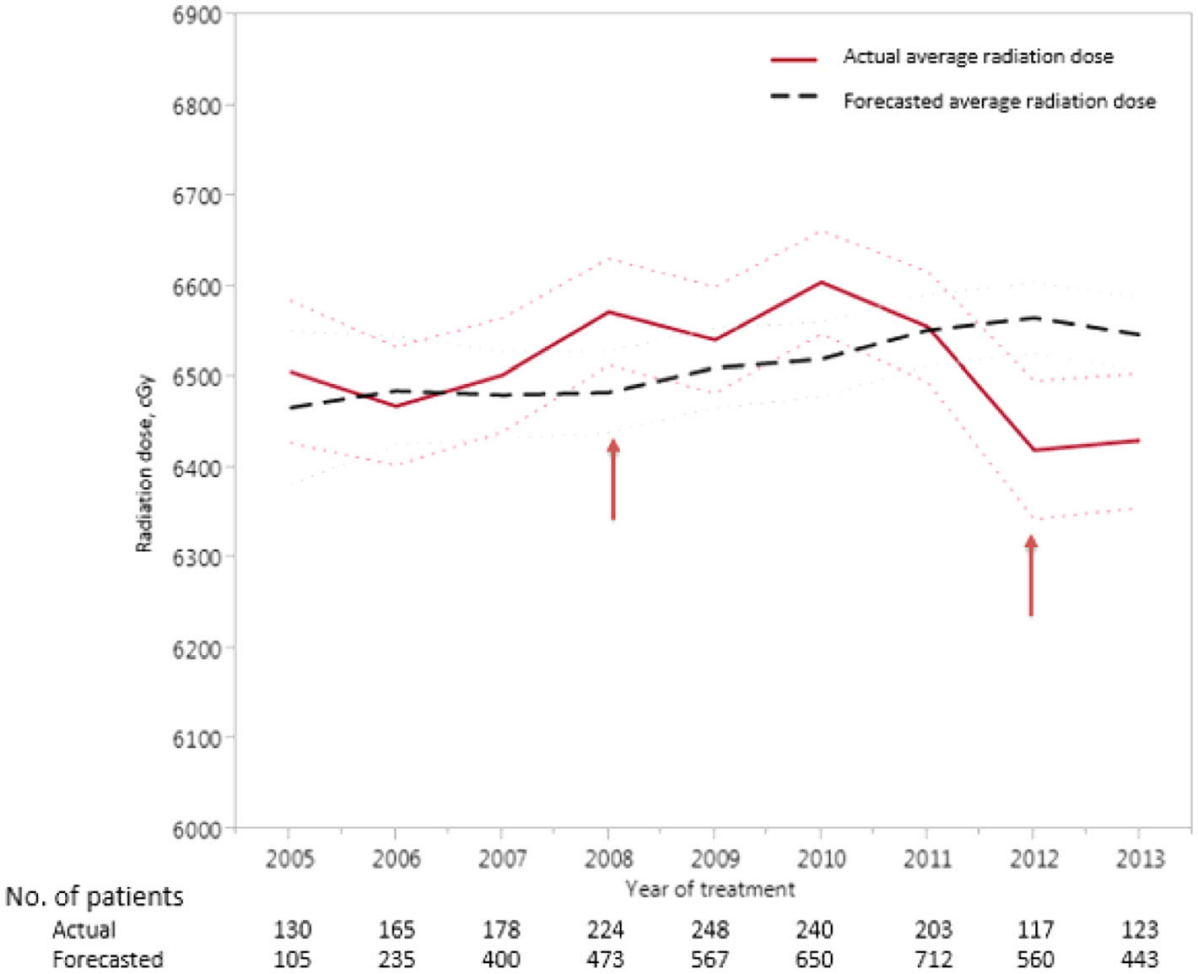
Actual average dose over time (solid line) versus the forecasted averaged dose (long dashed line) in the NODA network. The light dashed lines 95% confidence intervals. Arrows indicate years in which there were significant differences between the actual and forecasted average doses for a given year

Using a rolling three-year average and adjusting for patient age and comorbidities, an increase in average radiation dose would have been first detected in 2008 had the NODA network been monitoring radiation dose in this lung cohort (2005–2007: 64.6 Gy, 95% CI [64.4–65.3 Gy] vs 2008: 65.5 Gy 95% CI [65.1–66.3 Gy], adjusted *p* = 0.0084) (Fig. 6). A decrease in average radiation dose would have been first detected in 2012 (2009–2011: 65.6 Gy, 95% CI [65.3–66.0 Gy] vs 2012: 64.2 Gy 95% CI [63.4–65.0 Gy], adjusted *p* = 0.0004).

## Discussion

Our analyses show that the use of dose-escalated radiation (>70 Gy) in locally advanced NSCLCa increased in the U.S. between 2004 and 2010. This finding is consistent with our *a priori* hypothesis that “radiation dose creep” occurred in the absence of level I evidence, since no randomized comparisons of standard versus dose-escalated radiation were formally published over this period. Single-arm studies published in the 2000s suggested that dose-escalation of up to 74Gy in 2 Gy fractions was safe and effective [10–13]. Questionnaire-based surveys show that the evidence was compelling enough to already have changed practice by the mid-2000s, particularly for physicians who considered themselves thoracic specialists [14]. The use of escalating radiation doses in this cohort likely reflects in part disappointing local control outcomes demonstrated with the standard of care. When outcomes are poor, providers may be more open to deviating from standards of care. Even after the results of RTOG 0617 were communicated, it is clear that the radiation oncology community did not fully re-embrace the 60Gy treatment paradigm. While utilization of doses >70Gy declined to its lowest levels in 2012, our data suggests that less than half of patients were treated using low-standard doses (≥59 Gy to <64Gy) in 2012 and 2013. The perception that “more is better” appears to have persisted. Indeed, there is a growing interest in whether intermediate doses (64Gy < dose <74 Gy) might achieve the benefits of dose escalation without the excess costs [15]. Of course, the complex interplay of hypoxic [16], immune [17] and other in vivo responses to radiation and our limited understanding of dose-response of normal tissues make the clinic effects of dose-escalation hard to predict and possibly counter-intuitive.

We also observed that the use of dose-escalation decreased between 2010 and 2013. The preliminary findings of RTOG 0617 were first presented at the annual meeting of ASTRO in 2011 [18]. The first peer-reviewed manuscript was published in 2015 [4]. It is remarkable that the rate of dose-escalation declined by 2012 to levels observed in 2004, based on abstracts alone. In some ways, the rapidity of response is re-assuring, but raises the question: what would have been the consequences of dose creep for these patients had RTOG 0617 been delayed or never performed?

Finally, we demonstrated that an increasing average radiation dose in this cohort could have been detected by 2008 within the smaller NODA network. This result suggests that a national infrastructure for monitoring cohort-specific radiation dosing patterns already exists. Most centers in the U.S. now participate in the CoC’s credentialing program so are already abstracting information on radiation dose. Thus, the CoC, SEER and many other programs that aggregate cancer registry data internationally could feasibly monitor dose to identify cohorts for which dose creep is occurring. In addition, our demonstration used a simple forecasting approach. More sophisticated analytical approaches could provide greater and timelier information [19].

This study has important limitations. First, neither the NODA nor the NCDB captures a statistically representative portion of the U.S. cancer population. While the NCDB captures approximately 70% of cancer cases, diagnoses from small and rural facilities may be under-represented. Second, the absolute estimates for the utilization of dose-escalated therapy varied between the NODA and the NCDB analyses. In contrast, the NODA and the NCDB generated similar estimates for the utilization of low-standard dose over the same time period. This discrepancy may reflect differences in the characteristics of the institutions represented within each dataset and challenges that registrars have in interpreting radiation summaries of patients who have both a regional and a boost treatment, which is more common in patients receiving dose-escalated therapy. The NODA likely contains more accurate dose data, but it lacks the representativeness of the NCDB. Given that the NODA and NCDB have complementary strengths and weaknesses, the true absolute utilization rate of dose-escalation therapy over this period probably falls somewhere between the NODA and NCDB estimates. Third, our analyses do not adjust for radiation technique (e.g., 3D conformal vs IMRT). While the NCDB does have a field identifying “treatment modality”, the current options eligible for cancer registrars are not mutually exclusive. For example, a registrar can identify a treatment as 6MV photons or IMRT, though often a treatment is both. Because of this issue, we chose not to include this variable in our analyses. While increasing adoption of IMRT may in part explain the increasing utilization of dose-escalated radiation observed before 2011, it would not explain the decline after 2011.

## Conclusion

Patterns of radiation dosing in locally advanced NSCLCa changed in the U.S. from 2004 to 2013 and in the absence of level 1 evidence. These changes could have been identified using a simple radiation dose monitoring approach that uses data already aggregated from most cancer centers in the U.S.

## Abbreviations

ASTRO: American Society for Radiation Oncology
CoC: Commission on Cancer
NCDB: National Cancer Database
NODA: National Oncology Data Alliance
NSCLCa: Non small cell lung cancer
RTOG: Radiation Therapy Oncology Group
